# The Gray Institute ‘open’ high-content, fluorescence lifetime microscopes

**DOI:** 10.1111/jmi.12057

**Published:** 2013-06-12

**Authors:** PR BARBER, IDC TULLIS, GP PIERCE, RG NEWMAN, J PRENTICE, MI ROWLEY, DR MATTHEWS, SM AMEER-BEG, B VOJNOVIC

**Affiliations:** * Gray Institute for Radiation Oncology & Biology, Dept. Of Oncology, University of OxfordOxford, U.K.; † Institute for Mathematical and Molecular Biomedicine, King's College LondonLondon, U.K.; ‡ Richard Dimbleby Department of Cancer Research, New Hunts House, King's College LondonLondon, U.K.; § Randall Division of Cell and Molecular Biophysics, New Hunts House, King's College LondonLondon, U.K.; ‖ Now at The University of Queensland, Brisbane St LuciaAustralia

**Keywords:** FLIM, FRET, high-content microscopy, TCSPC, tissue microarray

## Abstract

We describe a microscopy design methodology and details of microscopes built to this ‘open’ design approach. These demonstrate the first implementation of time-domain fluorescence microscopy in a flexible automated platform with the ability to ease the transition of this and other advanced microscopy techniques from development to use in routine biology applications. This approach allows easy expansion and modification of the platform capabilities, as it moves away from the use of a commercial, monolithic, microscope body to small, commercial off-the-shelf and custom made modular components. Drawings and diagrams of our microscopes have been made available under an open license for noncommercial use at http://users.ox.ac.uk/~atdgroup. Several automated high-content fluorescence microscope implementations have been constructed with this design framework and optimized for specific applications with multiwell plates and tissue microarrays. In particular, three platforms incorporate time-domain FLIM via time-correlated single photon counting in an automated fashion. We also present data from experiments performed on these platforms highlighting their automated wide-field and laser scanning capabilities designed for high-content microscopy. Devices using these designs also form radiation-beam ‘end-stations’ at Oxford and Surrey Universities, showing the versatility and extendibility of this approach.

## Introduction

One of the most highly resolved optical imaging techniques for the study of *live* cells is fluorescence microscopy and this remains the mainstay for imaging intact cells and other biological samples. High-content information using automated fluorescence microscopes and large numbers of biological cells is required for many assays to counter the *biological* noise of the ensemble, for example the study of protein–protein interactions (Hu & Kerppola, 2003) and is essential where the aim is the detection of rare events, such as performing gene knock-down and RNAi assays (Neumann *et al*., 2006). Automated fluorescence microscopes designed for such experiments are therefore crucial to such assays and necessarily must be able to handle a large data throughput (the high-resolution scanning of a tissue microarray (TMA) or a 384-well plate of live cells may require the acquisition of more than 1 billion pixels of information). It is also important that, for whole assay automation, any microscopy imaging system developed is compatible with robot-assisted preparation of samples in terms of its physical arrangement (e.g. an inverted microscope geometry with simple access to the sample and/or minimal mechanical parts above the sample position).

With this in mind, the development of an ‘Open’ automated fluorescence microscopy platform, in which significant design aspects are not hidden, is very desirable in a research environment. It is further desirable that these platforms use readily obtainable components as far as possible, in conjunction with a minimum of specialised ‘in-house’ developed subassemblies. Where the latter are essential, these should be implemented to ‘open’ standards and complemented by mature and sufficiently advanced commercial products (cameras, detectors, lenses, etc.). As well as being able to meet the above criteria, such a system should also be able to accommodate novel techniques, which can be implemented and made available for routine biological use much more quickly than commercial companies can release a new product based on novel technology. The problems associated with relying solely on the commercial development of products is not just one of time, but this route is by no means guaranteed to happen at all, it may be expensive when it does happen and users may then be reliant on a specific company for support.

The commercial route has its place, of course, as operating microscopes of this type often requires support from technical personnel and, on a large scale, companies are best placed to provide this long-term support. The advantages of in-house development are clear when flexibility is required to perform new and varied experiments. Furthermore, the knowledge gained in development remains in-house, and includes device capabilities and limitations, such that the microscope is no longer a ‘black box’. Once a system has been installed, experiment flexibility is also highly desirable in a University research environment (as opposed to an industrial screening programme) and allows the use of different imaging modalities, such as time- and polarization-resolved imaging that have been included in our platform designs. The performance of an in-house system can be optimized for the local experiments and system components added as required. The platform must also provide quantitative data for bioinformatics and have the ability to optimize, often on a daily basis, what information is exported and in the most appropriate format. In-house developed systems are therefore optimal in many circumstances. By publishing and releasing details of our systems, with drawings, circuit diagrams and source code for custom items, we hope that other users can take a speedier route to in-house developed and maintained high-content microscope platforms.

Recently, an open-source software framework for driving such microscopes, Micromanager,^1^ has come to fruition (Edelstein *et al*., 2010). We chose, at an early stage in our platform development, not to use this framework but instead to develop our own. At that time, Micromanager did not support the devices we required and tests showed that it had stability problems. Device support for Micromanager and stability has improved and it would be possible in the future for our framework software to support the open-source Micromanager drivers. The two are thus not mutually exclusive. We stress that the decision to develop the in-house framework was driven by the need for greater flexibility and less dependence on third parties. Our own experience has shown that software under in-house control could be written to have the features desired of these platforms; we could not be sure that Micromanager could meet those needs. Other microscope acquisition programs are able to provide some automation through macro languages and similar approaches.^2^ These environments are not suitable for the development of the novel applications and functions that are described in this paper which require direct interfacing with time critical hardware and the use of manufacturers’ software development kits.

In particular, our implementations of automated platforms have been designed and constructed to incorporate fluorescence lifetime imaging (FLIM) and have been optically optimized for time-domain FLIM performed by time-correlated single photon counting (TCSPC), demonstrating the first implementation of this type whereas previously published systems have incorporated frequency-domain FLIM (e.g. Esposito *et al*., 2007). The optical path has been simplified to be more suitable for short laser pulses and provides high light throughput by minimizing optical paths and reflections from surfaces. The inclusion of FLIM is important as it is the gold standard method for the detection of protein–protein interactions via Förster resonance energy transfer (FRET; Becker *et al*., 2001) and is therefore particularly significant for high-content proteomic studies.

In particular, we have combined a laser scanning arrangement that is easily registered with camera-based imaging. This allows fast, sequential and automated operation with a conventional wide-field camera, using multiple fluorescence ‘cubes’ in combination with laser-scanning, or laser-scanning confocal, FLIM. A specific advantage of the system described here is the combination of laser, camera and stage scanning together for both micro and meso-imaging. Although similar arrangements have been recently made available commercially, (e.g. Prior,^3^ Leica^4^) we are better able to improve and change the system as required for specific applications. The switching speed between modes (wide-field fluorescence, laser scanning, etc.) is faster than in many commercial microscopes and is achieved through the use of a customized optical path switch and a cube changing unit; these modify the optical path, switching between modes in typically less than one second. To ensure that quantitative fluorescence microscopy can be performed over extended periods of time, and results compared between experiments performed weeks apart, the fluorescence excitation path includes an excitation optical power monitor that measures the illumination power of *every* fluorescence exposure. This provides us with the ability to measure long-term excitation stability. Furthermore the sample illumination offers extremely ‘flat’, i.e. even, illumination across the sample field, with less than ±2% variation across the imaged field.

In this paper, a general overview of a complete ‘open’ high-content FLIM microscope is provided and details are given on specific custom-made components, where space allows, and in the Supporting Information, where more detailed descriptions are provided. Furthermore, aspects of some custom designed parts are described online, where appropriate, on our group Website^5^ and detailed CAD drawings (SolidWorks,^6^ Waltham, Massachusetts, USA) can be made available to interested parties. To date we have constructed six systems; although the assemblies vary significantly, they share much of their hardware designs. The control software is more generally applicable and currently drives an additional four microscopes, based on commercial Nikon microscope bodies. This reuse of the software enables us to build up a suite of microscopes that all have a common interface but with varying mixes of in-house and commercial hardware, and varying levels of automation.

In the following sections the custom hardware components are described, followed by descriptions of the major software components. The applications section demonstrates how different combinations of hardware have been used for practical purposes since completion and details of the two principal systems named ‘Abbe’ and ‘Galileo’, installed at The Gray Institute, University of Oxford, and at The Randall Division, King's College London are presented here.

## Hardware development

**Figure 1:**
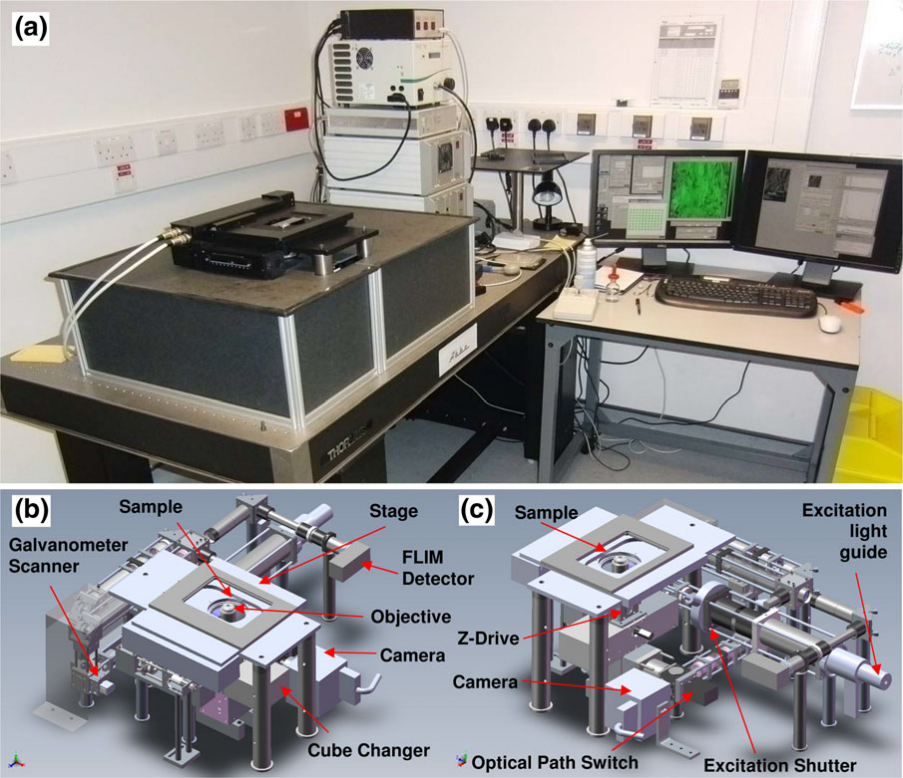
The Open Microscope ‘Abbe’. (a) External photograph with light shielding in place around the optical components. (b, c) 3-D rendered CAD of the internal optics.

The descriptions that follow relate principally to the ‘Abbe’ system is pictured in Figure 1 along with the 3-D rendered CAD output. The construction mainly consists of an optical cage framework (30- and 60-mm cage systems, Thorlabs Ltd., Ely, UK) with some custom made assemblies where extra stability or construction flexibility was required. The inverted fluorescence microscope arrangement is employed with no components above the sample stage so as to make it compatible with sample preparation robots (e.g. the PerkinElmer JANUS^7^). The systems occupy approximately 1 m^3^, with a bench footprint of ∼800 × 700 mm, including electronic modules and the computer housing, in addition to space for operator seating.

The main construction links together the following major commercial components: a fluorescence excitation illuminator (Lumen 200, Prior Ltd, Cambridge, UK); an optical shutter (VS25–52-ZM1, Uniblitz, Rochester, New York, USA); a white-light continuum laser (SC450-M, 80 MHz rep. rate, 4 ps pulse width, 450–2400 nm, Fianium Ltd, Hamble, UK); galvanometer-based mirror scanners (VM1000, GSI Group, Unterschleissheim, Germany); filters (Semrock Inc., Rochester, New York, USA) and filter cubes (TE2000 style, Nikon UK Ltd, Kingston Upon Thames, UK); piezo z-drive (Mipos 500 SG, PiezoSystem Jena GmbH, Jena, Germany); objective lens (e.g. 20x 0.75 NA S Fluor or 40x 0.9 NA S Fluor, Nikon UK Ltd.); motorised sample translation x-y stage (SCAN IM 100×100 with ITK Corvus Controller, Märzhäuser Wetzlar GmbH & Co. KG, Wetzlar, Germany); wide-field fluorescence camera (1.3 megapixel, ORCA-ERII, Hamamatsu Photonics UK Ltd, Welwyn Garden City, UK); photomultiplier time-resolved detector (PMH-100–0, Becker and Hickl GmbH, Berlin, Germany); TCSPC acquisition electronics (SPC-830, Becker and Hickl GmbH); and PC running Windows XP (Dual Pentium 3 GHz, 2 GB RAM, Scan Computers International Ltd, Bolton, UK). Numerous additional commercial components (lenses, mirrors etc.) have also been used and all are available off the shelf.

These commercial components are supplemented by in-house developed electromechanical devices controlled via an internal distributed I^2^C bus.^8^ This internal bus is derived from a single USB connection to the host computer. The I^2^C-driven subassembies include a communications hub, an optical path switch, a filter cube changer, laser scanners, an excitation power monitor and a coarse z-drive. These components are described in the following subsections and, in addition, the supporting and online information provide more details including circuit diagrams and printed circuit board layouts for these and other components and accessories.

The machining and construction cost of in-house developed components is no more than £6,500 (10,000 US$) which includes the framework, optical mounting and optics for the laser mounting, XY laser scanning system, fluorescence detection, cube slider, optical path switch and wide-field fluorescence illumination. The overall cost, including the galvanometer scanners, lenses, etc. is well within £11,000 (17,000 US$), which is significantly lower than that of commercial systems of comparable complexity and which are likely to be complemented by similar objectives, stages and stage controllers, cameras, etc, which inevitably raise overall cost. Commercial microscope bodies can be fitted with a number of output ports but these, in general, must be specified at time of ordering and cannot be retro-added when experimental requirements change. Commercial microscope bodies are engineered in a way to allow transportation and are often supplied in modular form, and their rugged construction is usually associated with a high degree of mechanical stability and low temperature drift. The stability of the Abbe system has been assessed over a period of 30 h when the unit was in a normal laboratory environment, with the ambient temperature varying by no more than a few degrees, by measuring the Z-drive autofocus position and X, Y image shift by crosscorrelation (see Supporting Information). The peak-to-peak drift in X, Y and Z was no more than 2 μm, with a standard deviation of less than 0.5 μm. Although microscopes with a monolithic body may have theoretical stabilities superior to this, it is our experience that, in practice, the overall stability is determined primarily by the particular stage inserts used and by the method of holding a sample (specimen slide, multiwell plate, etc.) in the stage insert. Tests with a Nikon Eclipse body, fitted with a similar stage, yielded very comparable drifts in the same environment.

### Fluorescence excitation path

**Figure 2:**
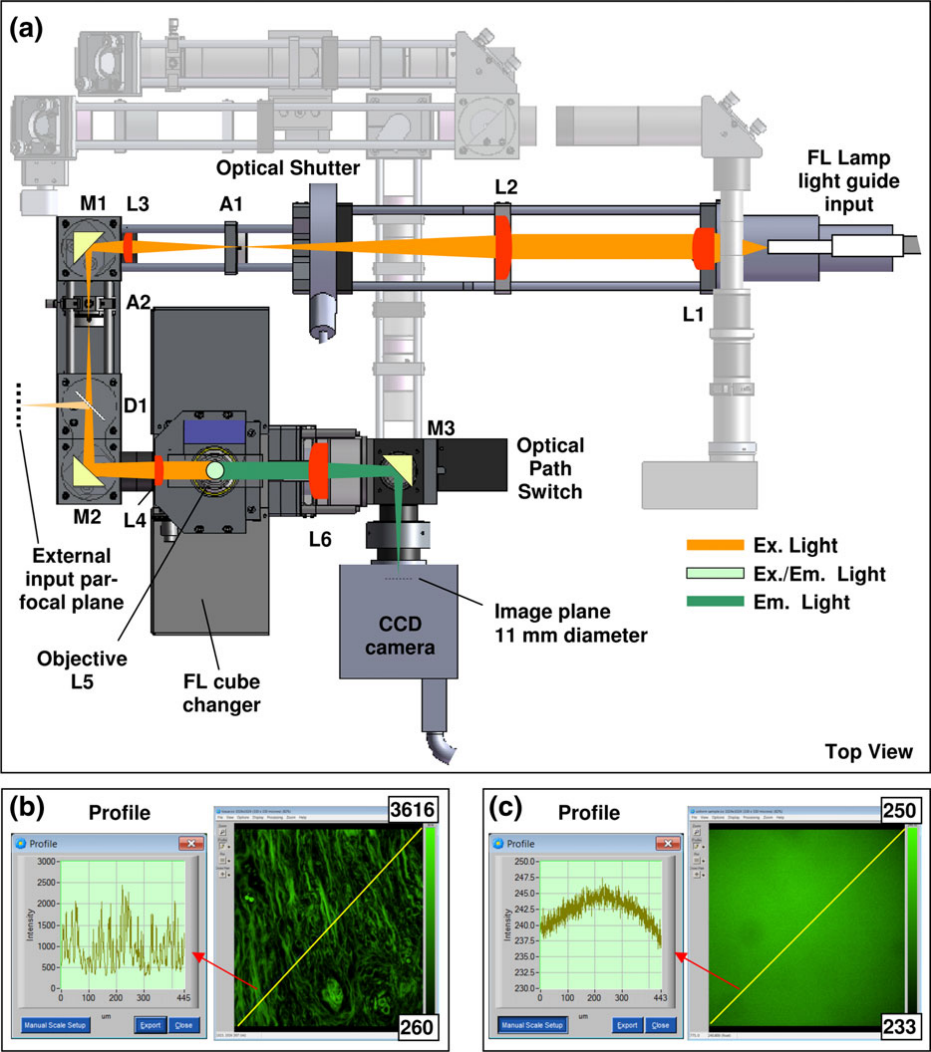
(a) Fluorescence (FL) excitation path for camera-based imaging: L, lens; A, aperture; M, mirror; D, dichroic mirror. The possibility for an additional excitation source is shown entering from the left via D1. (b) Example image of hematoxylin and eosin stained tissue taken with a FL cube for Cy2 with profile of a line drawn in yellow from bottom right to top left. (c) An average of 10 images of a uniform fluorescent sample at semi-random stage positions to even out tissue structure (normalized display bit depth) showing flat illumination to within 4% over the image (note the expanded vertical scale).

The fluorescence excitation path for wide-field camera-based imaging uses the conventional Köhler epi-illumination optical arrangement in a folded path as shown in Figure 2. This arrangement has been designed to provide a uniform and stable excitation field, a task made easier by the use of the Prior Lumen 200 source's liquid light-guide. Lenses (L1 and L2) collect and collimate the light from the guide through the fast optical shutter and the condenser aperture (A1). Lenses (L3 and L4) form the Köhler arrangement where the illuminated field can be adjusted with the field aperture (A2). The path is folded with mirrors M1 and M2. After L4, light is directed into the objective lens by the fluorescence filter cube (FL cube). Emission from the sample proceeds through the FL cube and is directed to the camera by a mirror and the optical path switch (*c.f*. Figures S2 and S6), focussed by a 200 mm tube lens (L6, Nikon CF 160 series, part #NT58–520, Edmund Optics Ltd, York, UK). The illumination field flatness (Fig. 2c) does not vary significantly with the range of 10x, 20x and 40x, S Fluor and LU Plan Fluor Nikon objectives in our possession. The illumination flatness is significantly superior to that provided by conventional mercury lamps and is primarily, but not exclusively the result of using a liquid-filled optical guide.

### Laser beam scanners

**Figure 3:**
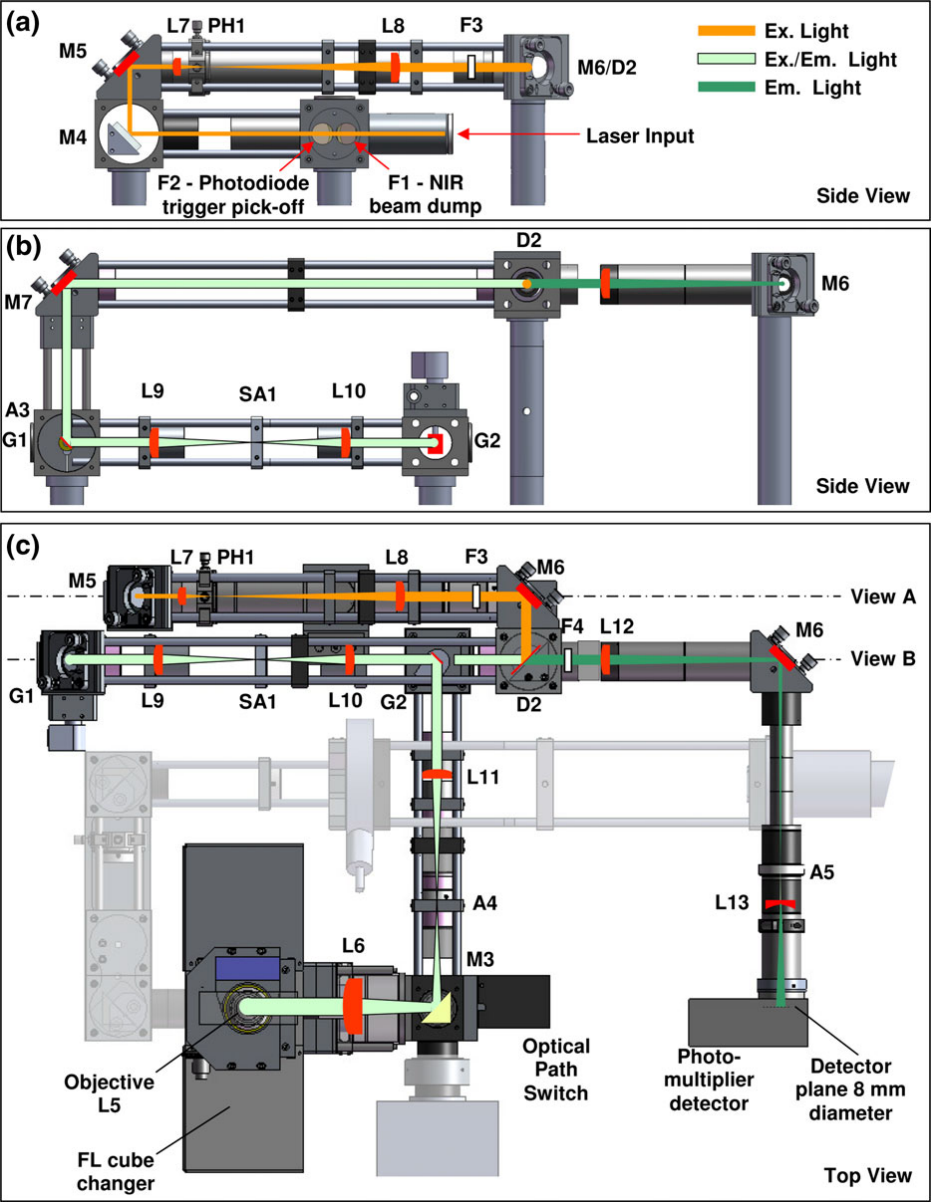
Schematic diagram of the laser scanning beam path (see text for description). L, lens; F, filter; PH, pinhole; A, aperture; SA, slit aperture; D, dichroic mirror; G, galvanometer. (a) Cut-away side view showing the laser beam conditioning optics at the back of the system. (b) View showing a cut-away side view of the beam scanning optics. (c) Top view of the whole system combining the optics from views (a) and (b) with the rest of the system including the photomultiplier arm.

Laser scanning microscopy requires a method of laser beam scanning, such that a collimated beam is pivoted on the back focal plane of the objective. In common with the majority of laser scanning microscopy, we use mirror-based galvanometer-driven scanners: these devices are straightforward to use and provide high light throughput. Two such scanners are required (for the X and Y directions) to be located at the first focal plane of the scan lens (L11 in Fig. 3) for optimal performance. In other systems, the scanners are often arranged to be both as close as possible to this point in a so-called ‘close-coupled’ arrangement, because two galvanometer-driven scanners cannot be physically at the same point.

**Figure 4:**
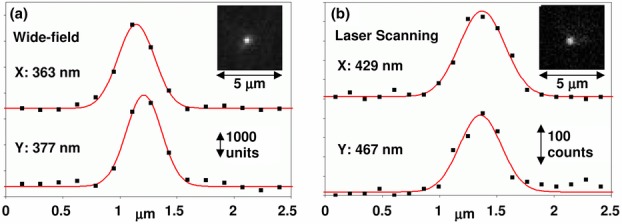
Optical resolution measurements from wide-field (a) and laser scanning (b) modes with filter sets for imaging FITC-like fluorophores. 100 nm fluorescent beads were prepared and imaged with a 40x 0.9 NA air objective according to standard protocols (Cole, Jinadasa and Brown, 2011). Pixel data in the X and Y dimensions are shown (squares) together with Gaussian profile fits (lines). The FWHM of the fitted profiles are shown. Measured point spread functions are slightly larger than the theoretical minimum (350 nm from imaging 100 nm beads at a wavelength of 500 nm) to achieve faster imaging with reasonable FLIM photon count. Fits were generated with the MetroloJ plugin for ImageJ (http://imagejdocu.tudor.lu/doku.php?id=plugin:analysis:metroloj:start).

To allow a greater optical aperture through the scanning system, we have chosen to link the scanners with an optical relay 4-f system (see Fig. 3). This decision introduces additional optics into the laser beam path and as a result may also compromise the beam quality and therefore the achievable image resolution. This is not such a problem considering the FLIM application where, in practice, the resolution is often reduced intentionally, through post-acquisition pixel binning, to obtain sufficient photon counts within each pixel or to provide a satisfactory time resolution with the available acquisition card memory, usually operated to provide a 256 × 256 pixel image with 256 time bins; any increase in pixel resolution comes at the expense of temporal resolution and *vice versa*. With careful optical design, however, a respectable point spread function is achieved, measured with 100 nm beads and a 40x 0.9 NA objective lens, to be 370 nm FWHM for wide-field and 450 nm FWHM for laser scanning (theoretical minimum FWHM ∼350 nm), as shown in Figure 4.

We also use a dedicated and optimized electronic scanner drive system that enables straightforward zooming about the central image point and exclusion of the non-linear parts of the scanner motion. More details are given online,^9^ including details of the mounting arrangement using the cage system,^10^ and in the section on the laser beam path, below.

### Supercontinuum laser integration

To provide flexibility for users to choose a range of fluorophores for laser scanning FLIM either several short-pulse lasers of differing wavelength must be provided, or wavelength bands from a single broadband source can be chosen, providing a more elegant solution. The range of supercontinuum fibre lasers that are currently available offer the opportunity to do the latter. The available wavelength range extends down to ∼450 nm (or lower, depending on laser model) and well into the infrared. These lasers also provide short pulses (∼4 ps) suitable for time-domain FLIM with a reasonably constant pulse shape over a range of powers. We select excitation and emission wavelengths with manually replaceable filters (Fig. 3, F3 and F4) and either a dichroic (Fig. 3, D2) or partially silvered mirror, though a motorised filter changer could also be incorporated. We have solved several engineering aspects associated with mounting these lasers and the exclusion of unwanted infrared light present in the beam; these are shown in Figure 3 (F1 and NIR beam dump) and more details are available online.^11^

### Laser scanning beam path

The beam path for laser scanning has been designed to offer an optimal compromise between beam and image quality and the number of optical components. Minimizing optical components has advantages in reducing light scatter and power loss while allowing simple control of reflections, often particularly troublesome when time-domain FLIM is used. Of course such reflections are present in other FLIM implementations, but are not immediately apparent. The beam path can be followed in the schematic diagram shown in Figure 3, as it emerges from the fibre coupling of the Fianium supercontinuum laser. Firstly, two near-infrared reflectors (‘extended hot’ mirrors, F1, F2), set at 45°, send the majority of unwanted infrared light to a beam dump and further to a fast photodiode that provides the reference trigger for the TCSPC electronics. The beam quality of supercontinuum lasers is in general not adequate, so the beam is passed through a spatial filter formed with two planoconvex lenses (35 and 150 mm focal lengths, L7 and L8), and a 20 μm pinhole (PH1, focal lengths and pinhole sizes vary between systems as the exiting laser beam diameter can vary between laser units). The recollimated beam then passes through a band pass filter (F3) to determine the excitation bandwidth and is directed by a beam splitter (D2) onto the scanning system (G1, G2). The in-house developed scanning arrangement uses two relay lens assemblies (L9, L10, 70 mm nominal focal length) in a 4f configuration, with a slit aperture (SA1) at the centre; this significantly reduces reflections and scatter. The beam diameter through the scanning system is intentionally arranged to be large (7.5 mm diameter) so as to minimize required scanner deflection angles and allow the use of a linear lens relay system rather than the more traditional, and superior, mirror relay arrangement (Amos, 1990). Our lens relay system offers good performance over ±2.5° scan angles using off-the-shelf Thorlabs achromatic and meniscus lenses.^12^ A scan lens, 100 mm focal length (L11) forms an intermediate image plane at an aperture (A4), such that when coupled with the 200 mm focal length tube lens (L6), the beam diameter is increased to 15 mm. This beam diameter is more than adequate to fill the back aperture of the majority of high magnification objectives. The use of small scan angles and a relatively long focal length scan lens allows us to use a simple pair of achromat lenses for this purpose.

**Figure 5:**
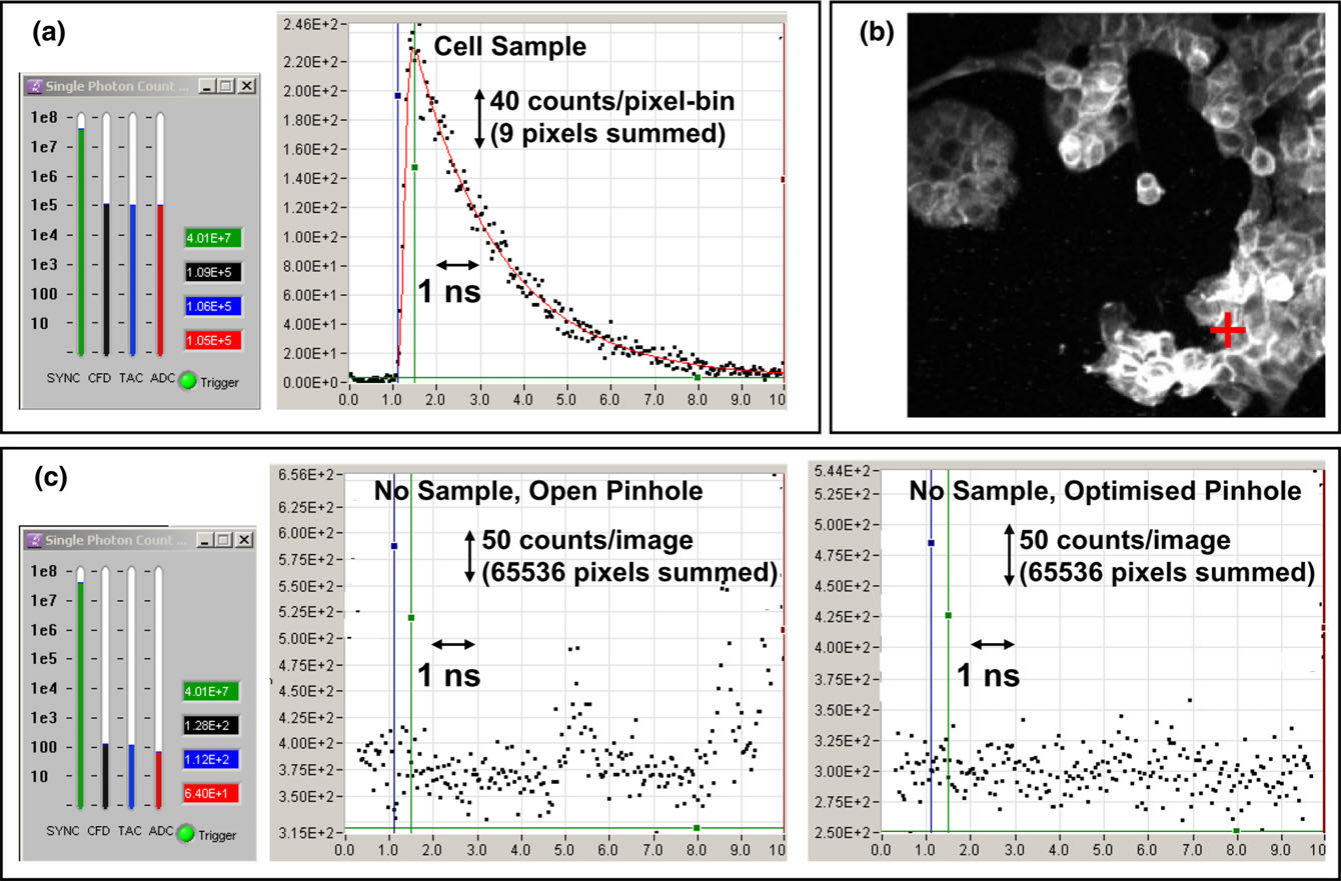
Comparison of FLIM signal versus background counts. (a) User interface showing a typical imaging photon count rate of approximately 10^5^ per second (at the CFD, TAC and ADC stages of the TCSPC electronics) and the transient signal from a 3 × 3-pixel binned area of an image of A431 human epithelial carcinoma cells expressing cdc42-GFP. (b) The image produced with the location of the highlighted 3 × 3 area (red cross). (c) With no sample present, and using the same excitation intensity, the count rates are approximately 100 per second and transients accumulated from all pixels in an image (65,536 pixels) show the background signal structure with the confocal pinhole (A5 in Figure 3) fully open (showing some residual optical reflections) and optimally sized. (Blue and green lines on the graphs are cursors marking detected excitation time extremes.)

The FLIM performance and excellent signal-to-spurious signal ratio that results from this optical arrangement is demonstrated in Figure 5. Figure 5a shows a typical average imaging count rate of 10^5^ counts/s that when combined with the ‘normal’ scan speed (40 μs pixel dwell time and 3 s scan duration including flyback time) results in 4 counts per pixel per scan on average. Typically counts are accumulated over 100 scans (total imaging time 5 min) such that the count per pixel is around 400 (3,600 per 3×3 pixel bin). A transient from 3×3 pixel bin is shown, from a bright part of the image where around 10^4^ counts have accumulated and have been distributed amongst 256 time bins (a lifetime of ∼2 ns therefore results in a peak amplitude near 200 counts/bin). This is sufficient to determine a monoexponential lifetime to within a few percent and to explore biexponential kinetics (Barber *et al*., 2005).

Using a test sample from a realistic experimental biological system the signal-to-spurious-reflection ratio based on count rate can be assessed. With reference to Figure 5(c), a background count, with no sample, of 300 counts/time-bin has a standard deviation of 17 counts/time-bin (approximately 2.6 × 10^−4^ counts/time-bin/pixel). Comparing this with a typical peak signal, acquired under similar excitation intensity, 27 counts/time-bin/pixel are obtained (250 counts/time-bin for a 3 × 3 pixel-bin, Fig. 5a). The signal-to-reflection ratio can thus be estimated to be at least 100,000:1 with an optimized confocal pinhole.

The FLIM instrument response function (IRF) is single-peaked with a FWHM of <200 ps. A low amplitude tail (<1% of peak value), extending to 1 ns is also observed. The observed IRF is similar to that reported for systems that use a metal-dynode photomultiplier detector (Becker, 2005), the tail being caused by photomultiplier after pulsing (Coates, 1973; Torre *et al*., 1983).

### Z-drive

Automated microscopes require computer controlled fine focus adjustment to account for variations in the desired focal plane across the sample, particularly when multi-well plates are used. Piezoelectric focus drives with 200 or 400 μm closed-loop objective travel (Mipos SG, PiezoSystems Jena) are used; this travel range is sufficient for experiments with standard multiwell plates or microscope slides. We have implemented an image-based autofocus algorithm used in conjunction with this drive, (see Supporting Information) using a spatial frequency-derived focus measure and a Fibonacci series based search algorithm (Kiefer, 1953). However, when nonstandard samples are used, when the focal plane lies outside the range of the piezo-objective drive, the absolute position of the objective must be changed although still retaining the fine adjustment piezo-system range of motion. An additional requirement is imposed by the initialization procedure for the X–Y stage, which usually requires motion to the extremes of the X–Y travel, taking the objective outside the normal operating range and potentially in danger of collision with the surround of the plate-supporting stage insert that in turn supports the sample. The objective must therefore be lowered (in our inverted configuration) some millimetres to avoid collision. A ‘coarse’ Z-drive with a DC motor-encoder position servo controller satisfies both these requirements and has been implemented and has an 8 mm travel with a speed of 0.2 mm s^−1^ and an ∼1 μm accuracy and repeatability. Figure S2 shows how this works with the piezo-z-drive for the desired result.

### Excitation intensity monitor

One of the important features that is easily implemented in such an ‘open’ framework is the provision of an excitation intensity monitor, particularly useful when quantitative fluorescence microscopy is required. This device enables monitoring of the excitation power at all times, when in wide-field or laser scanning mode, making it easy to compare and normalize experimental results. It offers an additional level of quality control and provides means to remove uncertainties associated with the illumination such as excitation source and optical alignment and sample staining efficacy; these are in general uncontrolled in traditional epifluorescence or laser scanning microscopy. Individual excitation monitors are easily calibrated against one another using a large area photodiode or power meter allowing comparison between microscopes in an additional level of standardisation.

The timing and duration of the power measurement must occur during the imaging exposure. This uses a software thread in the main program that is started simultaneously with acquisition. The thread contains an appropriate programmable delay to synchronize the measurement relative to the acquisition with an accuracy of a few milliseconds. The time required for digitization is of the order of 60 ms, and therefore the power associated with exposures of typically less than 100 ms cannot be measured. The excitation monitor uses a photodiode to monitor a partial optical reflection of the excitation light (Fig. S4). The photodiode output is passed through a logarithmic amplifier so that the very wide range (sub-nW to mW) can be processed before digitization.

### Fluorescence filter cube changing unit

Wide-field fluorescence imaging is conventionally achieved using a ‘cube’ of optical filters suitably arranged to give the required wavelength discrimination (Ploem and Tanke, 1987). We employ cubes made to fit Nikon TE2000 microscopes and use motorised switching between them for different wavelength ranges with a filter cube changing unit. For simplicity of construction, we opted for a linear arrangement of cubes. This has the disadvantage that only three cube positions can be implemented; however, we have found this sufficient for most high content imaging applications. Our unit employs a geared DC motor to drive the cube slider through a rack and pinion arrangement and a step-wise position feedback arrangement (Fig. S5). Cube changing occurs in around 1 s.

### Optical path switch

A motorised optical path switch is used in our platforms to guide light from the objective/tube lens to a camera or to the laser scanning arm for FLIM. This uses a kinematically mounted corner reflector prism, driven by a microstepping motor that offers position changing in hundreds of milliseconds. High level software controls this and keeps track of which device (camera, etc.) is on which optical port (Fig. S6).

## Software development

**Figure 6:**
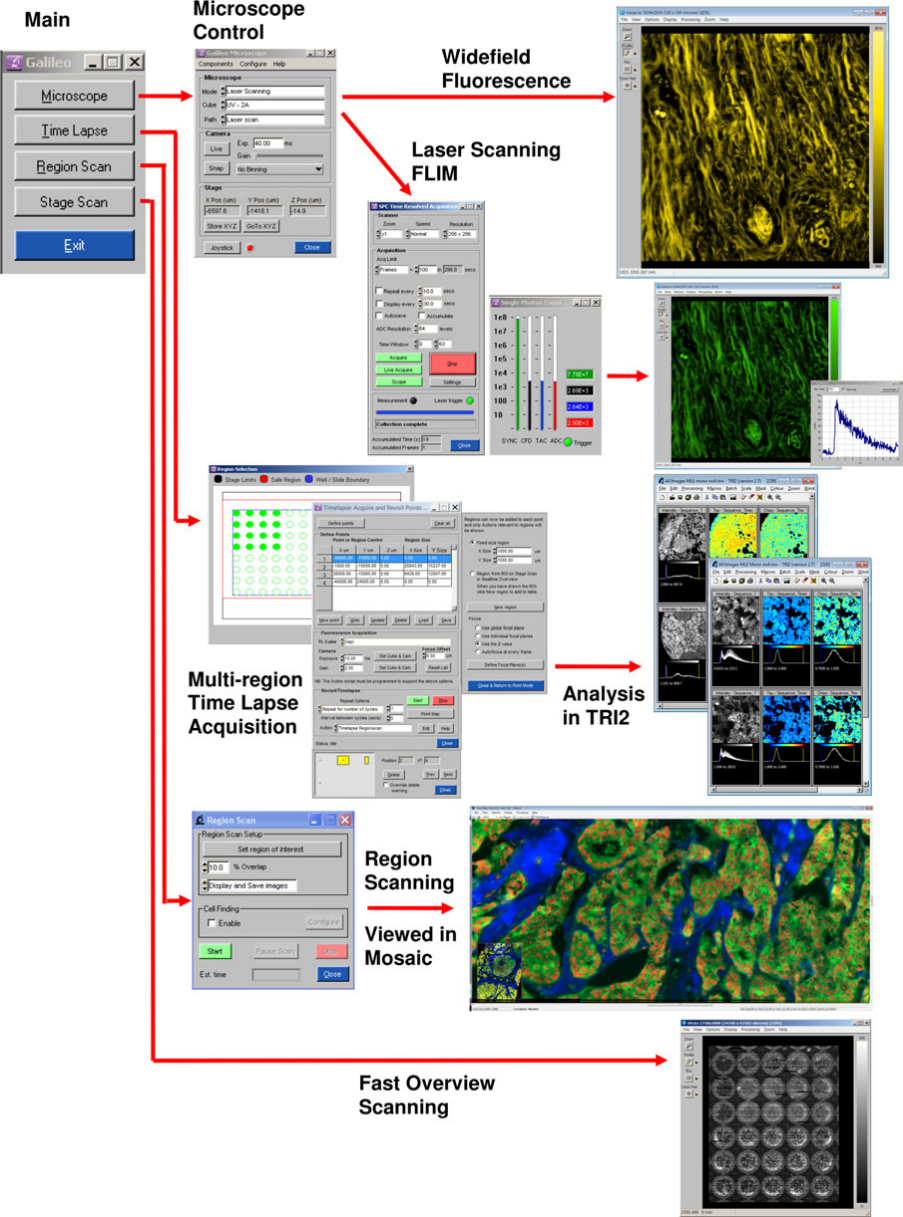
Schematic diagram showing the main functions of the Abbe microscope with associated software panels. From top to bottom: General microscope control with fluorescence wide-field and laser scanning FLIM; Multiregion time-lapse acquisition of multiwell plates and tissues resulting in batch analysis with the TRI2 program; Region scanning by tiling multiple fields and viewed in the Mosaic program; Fast overview of the sample by ‘stage scanning’.

Software is the ‘glue’ that empowers the hardware components to behave as an integrated imaging platform. This software ties together disparate hardware components, be they custom built or from an OEM supplier, to perform high-level functions and experiment protocols. The main capabilities of the current platforms are outlined in Figure 6 where the principal software panels are shown, as described in the following sections.

The software interfaces to all the controllable hardware components and keeps metadata on those that are not controllable. It handles the automated acquisition and saving of images and data as well as providing novel functions to locate regions of interest or the well of a multiwell plate, increasing the practical flexibility of platform use. The software remains modular to enable software ‘packages’ for different microscopes with different hardware and is object-oriented such that multiple devices of the same type may exist on one system. The integrated control of photon counting electronics and laser scanners allows automated FLIM via TCSPC and time lapse acquisition of cells, tissue and in vivo.

Especially novel aspects of the software include scriptable multipoint time-lapse and high-resolution large-area region scanning by image tiling, and these are described below. In addition, we have developed stand alone programs that provide postprocessing, such as ‘TRI2’ (Barber *et al*., 2009; Rowley *et al*., 2011) that provides time-resolved lifetime fitting and colour image unmixing, and ‘Mosaic’ which is able to stitch and blend image tiles for the exploration of arbitrarily sized regions or the export of mosaics at full resolution. More details are, again, provided on our Website.

### Python time-lapse scripting

Although the main program is written in ‘C’, the programming language Python^13^ has been embedded within it. This allows Python scripts to be called by the ‘C’ code that control the actions of the platform through custom classes that extend the standard Python language. Importantly, this has been used to form a generic and flexible multipoint time-lapse feature, allowing user-customization at run time. This makes the system flexible in the face of changing user needs, without the need for a complete recompilation of the program nor experience with ‘C’ programming.

### Region scanning and mosaicing

Region scanning allows large regions of tissue, or cell dishes, to be imaged at the platforms native resolution with a given objective lens. This is achieved by sequentially stepping the stage (i.e. sample) across the objective and acquiring one or more camera images at each step. The result is thus a large number of images that overlap slightly (typically by 10% of their XY dimensions) and together cover the entire region of interest. The region scanning function can also be called from a time-lapse script to form a region scan of each core of a TMA, for example, with a 4 × 4 mosaic.

We have previously published a method for stitching and blending such a set of images together into a complete mosaic (Rankov *et al*., 2005). This method has been extended and implemented in a stand-alone Windows program: ‘Mosaic’. This has been written in C# and enables the exploration of large mosaics, by intelligently loading into memory only the data that are required to form a user-selected view of the sample, at a user-selected resolution. Mosaic images can also be exported at full camera resolution, in the form of a single image, larger than a single camera field of view for additional processing elsewhere. This program uses no propriety code libraries and the source code and the Windows installer are available online.^14^

### Real-time overview and fast overview scan

The high-content platforms constructed thus far using this ‘open’ structure do not have any traditional eyepieces and only allow the use of one objective lens at a time (i.e. there is no ‘nosepiece’ that allows quick objective changes). These decisions were made for numerous reasons, not least because the fast piezoelectric-based focus control can only drive one objective. Of course, a direct consequence of this is that the user cannot use the conventional approach of exploring the sample at low magnification to find the specific location of interest and image this at high magnification. To overcome this potential limitation, two closely related novel features have been introduced, which we have named the ‘real-time overview’ and the ‘fast overview stage-scan’.

The real-time overview keeps a low-resolution copy of every image acquired from the camera along with its stage position, which it uses to place these image copies on a screen canvas such that they together form a representation of the sample. A single cross is also placed on this canvas indicating the current stage position in relation to the images. The result is a record of where the user has visited on the sample and a rough map of the sample is built up as the user explores it. Any coordinates of this map may be clicked to revisit any position, and an indication of what part of the sample is currently being viewed.

This feature has been extended to form the fast overview stage scan by letting the software explore the sample, in a systematic way, to build up the rough map of the sample. This is achieved by instructing the stage to perform a raster scan of the sample, with an appropriate speed and line spacing. During this scan the camera acquires images at a low-resolution and at high speed; with the ORCA camera in the 8 × 8 binning mode, around 40 frames per second can be achieved. In this manner a sample region 30 × 20 mm can be mapped in a few minutes. This mapping significantly increases the speed with which TMA cores or other sample areas of interest can be identified.

## Applications

In this section we briefly describe some of the applications to which we have applied our open microscopy platforms, several of which have been previously published (e.g. Patel *et al*., 2011; Carlin *et al*., 2011; Fruhwirth *et al*., 2011; Waterhouse *et al*., 2011). These platforms excel at reducing the workload of the user when numerous time-consuming FLIM images are to be acquired. This is most effective when the sample is a TMA or a 96- or 384-well plate. In these scenarios the user may use the fast overview scan to map the sample and then build up a list of regions to image (stage X, Y and focus Z coordinates), using conventional wide-field fluorescence microscopy. This may take less than 30 min to perform. Alternatively, if a multiwell plate is used, the user may allow the computer to generate a list of points based, for instance, on the well centres. A script can then be chosen that may visit every point in the list, perform image-based autofocus, acquire wide-field images with all fluorescence cubes present and finally acquire a FLIM image. This process may take more than 8 hours to complete but during which the user does not need to be present. The progress of the microscope can usually be easily checked using the user's favourite remote computing method (e.g. Windows Remote Desktop Protocol).

**Figure 7:**
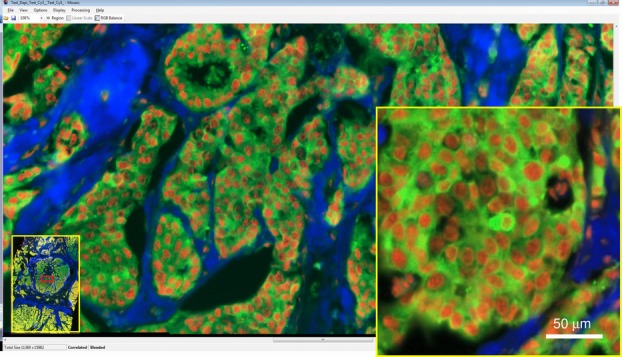
Multichannel image tiling with edge correlation and blending of breast tissue labelled with anti-ezrin IgG-Cy2 (Green) and anti-phospho PKCα (T250) IgG-Cy3 (Red), together with an a UV-2A cube filter set image (Blue). A 500 μm wide view from 3.3 mm wide 184 Mpixel image. The dataset was assembled and rendered in our Mosaic program with complete slide overview at bottom left and red rectangle of current view which is displayed as a full resolution image in the main display. Inset is a high-resolution view.

The detection of intermolecular FRET between directly labelled anti-ezrin IgG-Cy2 and anti-phospho Protein kinase C α (T250) IgG-Cy3 in breast tissues is the subject of recent research of our group and collaborators (Fruhwirth *et al*., 2011). In Figure 7 we show the membranous ezrin localization typical of invasive breast cancer through a large region scan of doubly stained tissue. The anti-phospho PKCα (T250) IgG cross-reacts with an 180 kDa non-PKC protein that is located mainly in the nucleus, giving rise to the nonspecific nuclear staining but also colocates with ezrin in breast cancer as determined by FRET (Ng *et al*., 2001).

**Figure 8:**
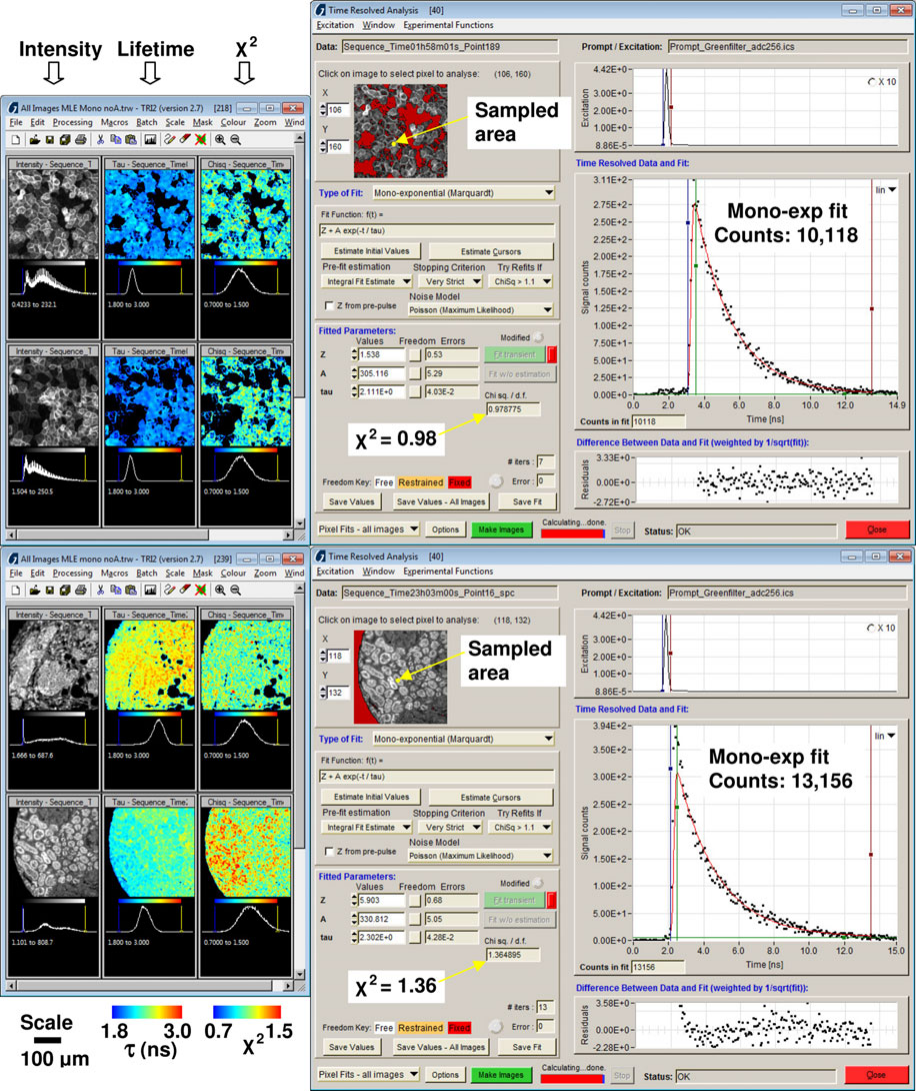
Examples of time-resolved imaging of fixed cells transfected with green fluorescent protein (GFP) and tissue sections stained with Cy3-conjugated antibodies. In this example, GFP has a clear monoexponential response whereas the tissue section images report a wider range of lifetimes with a multi-exponential response, probably because of variations in local fluorophore conditions and interfering fluorescence from endongenous species. In the lower panels, lifetime analysis of antibody tagged ezrin-Cy3 reveals FRET, via a reduction in lifetime in the lower tissue images, when colocated with PKCα-Alexa546.

Figure 8 shows lifetime analysis of a variation of the ezrin:PKCα experiment this time using antibody tagged ezrin-Cy3 and PKCα-Alexa546 (lower panels), shown below an example of the analysis of GFP fluorescence. Human epithelial carcinoma cells (A431) stably expressing cdc42-GFP were imaged by time-domain FLIM and screenshots from the TRI2 program show the clear monoexponential GFP response when prepared in this manner, compared to the more complex response from a TMA of paraffin embedded tissue.

As an example of published work, we performed a screen for small-molecule compounds that may interfere with a specific cancer-relevant cell signalling pathway (Fruhwirth *et al*., 2011). FRET was used as an indicator of protein-protein interaction Mst2-Raf1 which is significant in linking mitogenic and apoptotic pathways. A novel biosensor containing the two proteins and the FRET pair mGFP and mRFP1 was used to detect interference because of target compounds. Breast cancer cells were seeded into a 96-well plate. Compounds and controls were assigned to wells of the plate with all compounds tested in duplicate adjacent wells. Five regions were imaged from each well, two compounds were found to significantly change the biosensor response compared to the controls. Analysis was performed using in-house developed lifetime fitting software (TRI2).

Work involving primary antibodies in TMAs has also recently been published that highlights the use of these platforms towards personalized treatment for cancer by Kelleher *et al*. (2009). In addition, to demonstrate the flexibility of these in-house developed systems it is worth highlighting another recently published work by Matthews *et al*. (Matthews *et al*., 2010; Matthews *et al*., 2012) in which wide-field anisotropy capability has been included into one of the open microscopes through the addition of a polarization-resolved imager (Quadview, Photometrics, Tucson, Arizona, USA). This showed how long-term, unsupervised imaging could take place to reveal FRET in a biosensor in a large number of samples via anisotropy.

The ‘open’ microscope approach has also been used to construct two radiobiology ‘end-stations’ at the ends of radiation beam lines and enable fast radiation targeting and imaging of biological material (cells and tissues). The placement of advanced fluorescence imaging systems of the end of a radiation beam line opens up completely novel radiobiological studies, where the consequences of ionising radiation on biological materials can be studied *in situ*, at short times following irradiation.

One of the installations developed with the approach described here is located in the Wolfson Tower at Surrey University, UK (Merchant *et al*., 2012). In that case an ‘upright’ microscope arrangement was used, imaging cells with a water-dipping objective, where the cells are irradiated with a high energy particle beam (protons and other heavier ions) from below. Conventional microscope bodies are completely unacceptable in such a situation, where radiation beam control elements have to be combined with trans-illumination condenser optics (Kirkby *et al*., 2007) and where the objective must be moved to allow clearance for sample insertion (rather than the stage lowered, as in conventional microscopes).

The second end-station is installed at the Gray Institute linear accelerator facility (described online^15^), in the Department of Oncology, Oxford, which uses submicrosecond pulses of 6 MeV electrons to trigger DNA damage in mammalian cells. Signalling mechanisms active at short times (seconds) and longer (hours) involved in the recruitment of DNA repair proteins are studied, using multiple markers (at different wavelengths) and time-lapse microscopy. This arrangement uses a very similar ‘inverted’ microscope to that described here, but in addition is coupled to a robotic sample transfer system, controlled by complementary software tools integrated with the microscope software. The installation is housed in an environmental enclosure providing the necessary 37°C/5% CO_2_ environment appropriate for live-cell time-lapse imaging. Here, ionising radiation levels are very high, and necessitate extensive radiation shielding of the microscope, using lead. Once again, commercial microscope bodies could not be used, partially because of their footprint and difficult-to-shield shapes. Moreover, access to any optical components damaged by long-term exposure to ionising radiation would be difficult and probably impossible, particularly if these components are cemented into place.

## Conclusion

We present an ‘open’ microscopy platform design methodology and several physical implementations that allow advanced fluorescence microscopy techniques to be incorporated into automated biological assays. The design is described as ‘open’ for two reasons. Firstly, drawings and diagrams have been published on the internet under an open license for non-commercial use, so that other research departments may benefit. Secondly, it allows easy expansion and modification as it moves away from the use of a commercial, monolithic, microscope body. It uses readily available commercial optical components and a modular and extendable software framework.

Six such platforms are now in use within our group and collaborators, in Oxford University, King's College London and Surrey University. These platforms use combinations of hardware and software from the designs presented here. Three of these platforms incorporate time-domain FLIM via TCSPC implemented in an automated fashion. Previously published experiments, that contain data obtained with these devices, are highlighted here to show that imaging data can be obtained from multiwell plates in a time-lapse fashion and from tissue slides and tissue microarrays. Platforms using these designs also form radiation-beam ‘end-stations’ in Oxford and Surrey and show the versatility and extension of this approach in experimental systems not traditionally readily coupled with microscopy.

This work demonstrates the first implementation of time-domain FLIM in an automated platform and presents a novel and groundbreaking resource for the biological microscopy community that will help to bridge the gap between the development of advanced fluorescence microscopy techniques and their routine use by biologists.

## Footnotes

http://valelab.ucsf.edu/~MM/MMwiki/index.php/Micro-Manager

e.g. ImagePro: http://www.mediacy.com/index.aspx?page=Image_Pro_All_Prods, and Metamorph: http://www.metamorph.com

http://www.prioruk.com

http://www.leica-microsystems.com/products/light-microscopes/

http://users.ox.ac.uk/~atdgroup

http://www.solidworks.com/

http://www.perkinelmer.com/Catalog/Category/ID/Janus

“I^2^C bus specification and user manual”, http://www.nxp.com/acrobat/usermanuals/UM10204_3.pdf

http://users.ox.ac.uk/~atdgroup/technicalnotes/Opticalscannergalvanometercontroller.pdf

http://users.ox.ac.uk/~atdgroup/technicalnotes/Asimplemountforscannergalvanometer.pdf

http://users.ox.ac.uk/~atdgroup/technicalnotes/Fianiumlaseropticalheadmounting.pdf

http://users.ox.ac.uk/~atdgroup/technicalnotes/Opticallaserbeamscannerlensrelaysystem.pdf

http://www.python.org/

Source code: https://github.com/atdgroup/Mosaic and installer: http://users.ox.ac.uk/~atdgroup/software

http://users.ox.ac.uk/~atdgroup/radiation.shtml

## Supporting Information

Fig S1 USB1 CommunicationsHub Interface board. Designed to be rack mounted, it has connectors for equipment internal and external to the rack chassis.

Fig S2 The basic assembly of the inverted microscope: two blocks attach a corner reflector mirror to the base. A fluorescence cube assembly is placed on top of this and the sample imaging objective is screwed into a combined coarse and fine adjustment focus assembly.

Fig S3 (A, C) CAD reconstruction and photograph of the dual z-drive showing an objective lens mounted onto the piezodrive. This is all atop a platform driven by a PIC controlled motor via a simple mechanical arrangement. (B, D) Software panels for controlling the coarse drive and piezo-drive.

Fig S4 Excitation IntensityMonitor. (A) Completed viewwith cage mount ready for insertion into C4W cube. (B) Cover removed to reveal the custom compact PCB. (C) Software panel showing measured level and information about the calibration.

Fig S5 Fluorescence Filter Cube Changing Unit CAD reconstructions and photographs. (A) CAD view with transparent access covers. (B) Close-up of the cube holder (one cube of three in place). (C) Completed external view. (D) Cover removed to allow cube access.

Fig S6 (A) A view of the four-position motorised optical path selector from the input side. (B) Exploded CAD reconstruction.
