# Supplementary material for: The Gray Institute ‘open’ high-content, fluorescence lifetime microscopes
Source: J Microsc. 2013 Jun 12;251(2):154–67. doi: 10.1111/jmi.12057 (PMC3910159; doi:10.1111/jmi.12057)
Supplement: Fig S1 — USB1 CommunicationsHub Interface board. Designed to be rack mounted, it has connectors for equipment internal and external to the rack chassis. [file jmi0251-0154-sd1.doc]

# Supporting Information

## 1. Communications

Wide-field image data is supplied to the PC through a FireWire (IEEE1394), a separate USB2.0 or a CameraLink bus, depending on the specific camera(s) used. In addition, the sample stage driver uses an RS232 bus, adapted to be driven through a USB port with one of numerous RS232-USB in line converter modules that are available (e.g. UC-232A, ATEN UK Ltd, Slough, UK).

The computer communicates with the custom hardware via single USB cable using an in-house developed USB1 Communications Interface. A communications hub converts USB data into an I^2^C bus, distributed to other components in a star and/or daisy chain arrangement. The hub is based on the FTDI USB-FIFO interface assembly (DPL245, Future Technology Devices International Limited, Glasgow, UK) that includes an on-board Peripheral Interface Controller (PIC) microcontroller and is incorporated into a custom printed circuit board, as shown in Figure S1. PIC software to receive and interpret commands via the USB port and relay them to the I^2^C bus has been written and is available online[[1]](#footnote-2), with host PC code to send commands via the native D2XX FTDI driver (via the proprietary driver FTD2XX.dll from Future Technology Devices International Limited) or a virtual COM port. Although relatively slow, communication speeds through this hub are more than sufficient for the platform’s electromechanical control at around 100 kb/s if interconnecting cable lengths are kept to less than a few metres in length.

In addition to the I^2^C bus output, several parallel I/O lines from the PIC are left free and used for applications where direct logic communication is beneficial. One of these lines is used to trigger the shutter driver on the wide-field epi-fluorescence illumination path so that this action can be performed much faster than via the I^2^C bus. To operate the shutter in a robust and timely manner, a driver circuit has been designed[[2]](#footnote-3).

## 2. Z-Drive

The piezo focus device is mounted on the central tower of components that form the base for an inverted fluorescence microscope, as shown in Figure S2 and online[[3]](#footnote-4). This contains the objective lens mounted onto the coarse and fine Z-drives, the fluorescence cube (contained within a Fluorescence Filter Cube Changing Unit, see below) and a large 90 degree turning mirror that allow the optical path to be extended horizontally. A 200 mm focal length tube lens is mounted on the output side of this mirror block. The tube lens is closely followed by an optical path switch (see below) to direct the optical path to the camera or the laser-scanning path.

Full details of the servo controller for the coarse Z-drive can be found online[[4]](#footnote-5); this uses a PIC microcontroller that drives a geared DC motor/encoder assembly (part #333-118, Maxon Motor, Finchampstead, UK). Closed loop control and pulse-width modulation is used to control absolute position and approach speed respectively. The motor is belt-coupled to a micrometer fitted with a toothed gear which in turn drives the objective platform with ~1 μm accuracy and repeatability. The travel range of this arrangement, shown in Figure S3, is approximately 8 mm and the travel speed is around 0.2 mm/s. Fine focus, provided by the piezo focus drive, completes the required objective motion in a few tens of milliseconds.

## 3. Excitation Intensity Monitor

This light intensity monitor is based around a photodiode (Centronic OSD5-5T) which samples the intensity, at the rear aperture of the objective, of the laser scanning beam or the wide-field fluorescence excitation. Mounting has been engineered to be compatible with the Thorlabs cage system and measurements are made on the light sampled by a glass coverslip beam splitter placed in the beam path (see Figure S4). A dynamic range of about 6 decades is required to allow both wide-field and laser-scanning excitation to be measured. This range is achieved by using a logarithmic amplifier directly after the photodiode (Analog Devices AD8304). The output of the logarithmic amplifier is digitised to a 16 bit resolution and, once again, the I^2^C bus is used for data transfer. Diagrams, circuits and code can be found online[[5]](#footnote-6). The measured value is corrected in software for the objective back aperture size and for the excitation wavelength; the latter is determined from the specification of the particular filter set selected (laser scanning) or filter cube (wide-field), as defined in the system metadata. The photodiode responsivity (peaking at ~0.45 A/W) across the visible wavelength range is used to derive the sampled optical power; this in turn has been calibrated against power present at the back aperture of the objective.

## 4. Fluorescence Filter Cube Changing Unit

An embedded PIC (Microchip Technology Inc, Chandler, Arizona, USA) controls the motor and drives the required cube to the correct position. Miniature magnets below the cubes and Hall-effect sensors provide incremental rather than continuous position feedback to the PIC. Switching between adjacent positions takes ~1 second. This description is made clearer with reference to Figure S5, where CAD reconstructions and photographs are shown. Software code for the PIC controller and higher-level PC code are available online[[6]](#footnote-7), together with circuit diagrams, PCB layouts and CAD drawings for a 3-cube driver system. The arrangement is made compatible with Thorlabs 60 mm and 30 mm cage components to permit straightforward integration with the rest of the system.

## 5. Optical Path Switch

The optical path switch enables a 25 mm diameter optical path to be directed to one of four output ports (Figure S6). It is designed to fit the Thorlabs C4W cage system cube and directs the path using a 45 degree mirror prism. Motion is provided with a microstepping motor (M2-211-13-01, Lin Engineering, Morgan Hill, California, USA) with directly attached I^2^C board for control via our USB1 controller (see Figure S1). Switching is fast with the movement between adjacent positions occurring on the order of 200 ms. Details of assembly and alignment can be found online[[7]](#footnote-8), as can control software code.

***6. Stability Measurement***

Ambient temperature was measured by an EasyLog USB temperature logger. An H&E stained thin tissue section (<<10 μm) was used as a sample, which was imaged in epi-fluorescence mode with a filter cube for FITC with the 20x 0.75 NA objective. This was focused using the image auto-focus routine with a range of 20 μm and accuracy 0.5 μm. After a stabilisation period of one hour, 5 images were acquired (each auto-focussed) every half an hour for 30 hours. Z readings were taken from an average of the five auto-focus positions. X, Y shifts were taken by determining the sub-pixel shift in object position relative to the imaged field of view, with respect to the first image acquired, using the “phaseCorrelate” function of OpenCV ([www.opencv.org](http://www.opencv.org/)). Again, the five readings at each half hour were averaged. These data were assessed for maximum deviation and standard deviation from the mean over the 30 hour period.

The results are summarised in the table below:

|  | Maximum Deviation | Standard Deviation |
| --- | --- | --- |
| X | 1.91 μm | 0.41 μm |
| Y | 1.39 μm | 0.33 μm |
| Z | 1.68 μm | 0.44 μm |

## 7. Software

The software has been written using Visual Studio Express 2008 (Microsoft Inc., Redmond, Washington, USA) as the main development environment but relies on Labwindows/CVI libraries (National Instruments Corp., Austin, Texas, USA) and user interface components. Images are handled by the open source ‘FreeImage’ library[[8]](#footnote-9), upon which we have developed the ‘FreeImageAlgorithms’ library.

Many scientific hardware devices are controllable from software and by far the majority of the simpler devices are controlled via RS232 through a PC COM port. In some cases a USB serial converter is embedded in the device such that the final connection to the PC is USB. This is desirable as multiple USB devices are easier to accommodate on current PCs than multiple physical COM ports. If a USB serial converter is not embedded, we often choose to add one to remove the need for multiple physical COM ports. We use our own version of this arrangement in the USB1 Communications Interface (described above).

The microscope application software has been written in modular ‘C’ for easy assembly and development of multiple systems with different components such as: light sources, shutters, filters, objective and optical path selection, camera and FLIM detection. More details of the software architecture and libraries used can be found online[[9]](#footnote-10).

## 8. Python Time-Lapse Scripting

A wide variety of scripts are available and can be extended to image sequentially each point with a number of fluorescence cubes in turn, or to image with all cubes at every point in turn, for example. Additional extensions to Python allow for control of all aspects of the microscope, including FLIM, autofocus and region scanning. Of course, these methods are equally applicable to fixed samples where each stage position need only be visited once, when sample features do not change with time. Basic and essential time-lapse functions have been implemented in the main program, such as the storage and management of a list of (x,y,z) stage positions or regions (that may be larger than 1 field of view) to be visited and the time interval between each cycle of re-visits. The re-visiting of the points and the functions that are performed at each point are controlled by a Python script with several essential methods (aka program functions or routines) that must be defined within the script. These essential methods are as follows:

1. **OnStart()**: Defines what should be done before the time-lapse process starts
2. **OnAbort()**: Defines any functions that should be performed if the user aborts the time-lapse.
3. **OnCycleStart()**: The main program calls this method to start a re-visiting cycle through the stored stage positions (points). To be completely flexible, this method is required to call a function called microscope.VisitPoints() to start the cycle of re-visiting.
4. **OnNewPoint()**: Defines the functions to be performed at each stage position.

## 9. Image Based Autofocus

Maintaining an accurate focus on the sample is essential in microscopy. It is therefore highly desirable for an automated microscope to have some form of autofocus for situations where the user is not able to re-focus every region of interest on the sample and the focal plane cannot be simply estimated by an analytic function. Quite often it is sufficient to derive a planar linear fit to 3 focus points, as used by the region scan function, or a bi-quadratic surface fit to 7 points, as used for well plates.

We have implemented an autofocus algorithm that uses image spatial frequency content as the focus measure (via the FFT), and a Fibonacci series based search algorithm to find to optimal focus plane. This typically takes 13 images and approximately 5 seconds to achieve an accuracy of <1 μm from a search range of 100 μm. Usually the search distance is considerably less and much faster optimisation is obtained when performing autofocus on closely spaced regions on a sample.

## 10. Cell Preparation

Human epithelial carcinoma cells (A431) stably expressing cdc42-GFP were grown in Dulbecco’s modified Eagle’s medium containing 10% fetal calf serum. Cells were seeded on a glass coverslip (50000 cells/ml) and 48 hours later were fixed with 4% paraformaldehyde, treated with 1mg/ml NaBH_4_, then mounted with Mowiol containing DABCO (2.5%(w/v) 1,4-diazabicyclo[2.2.2]octane, VWR International Ltd, Lutterworth, UK) and kept at -20 degC. Data acquisition was performed at room temperature.

## 11. Tissue Micro-Array Preparation

The Hedley Atkins Breast Pathology Laboratory, originating in 1974, maintains and continues to accrue a unique resource of archived breast cancer material. It is a repository of well annotated formalin fixed paraffin embedded (FFPE) tumours. Cores from breast tumours have been used to create TMAs with 84 cores per slide, complete with template data linked to clinico-pathological information. Two 1.5 mm cores were obtained from individual blocks and re-embedded in microarray blocks. TMA blocks were cut 3mm thick.

TMAs sections were baked at 56^o^C then dewaxed using xylene (2 x 5min washes), followed by 2 x 2 min washes in absolute alcohol and 1 x 1 min washes in 70% ethanol and running water for 5 minutes. Heat medicated antigen retrieval was carried out by placing slides in a DAKO Pascal retrieval device, containing DAKO retrieval solution at pH6.0 for exactly 2 minutes at full pressure (22 psi). Once pressure was released, slides were cooled gently in running tap water, then transferred to deionised water. Sections were washed in PBS and incubated in fresh sodium borohydride (1mg/ml in PBS) for 10 minutes to quench background fluorescence. Three additional washes in PBS were followed by blocking using 2% BSA in TBS (filtered on ice) for 30 minutes. Excess BSA was drained and filtered antibody (diluted in filtered BSA) was then applied. Sections were incubated in darkness for 4 hours. Three PBS washes were followed by a final wash in deionised water. Coverslips were mounted using Mowiol containing DABCO (2.5%(w/v) 1,4-diazabicyclo[2.2.2]octane, VWR International Ltd) to prevent bleaching and allowed to solidify overnight at room temperature.

1. http://users.ox.ac.uk/~atdgroup/technicalnotes/USB1 communications interface for controlling instruments.pdf [↑](#footnote-ref-2)
2. http://users.ox.ac.uk/~atdgroup/technicalnotes/Uniblitz optical shutter driver.pdf [↑](#footnote-ref-3)
3. http://users.ox.ac.uk/~atdgroup/technicalnotes/An inverted fluorescence microscope assembly.pdf [↑](#footnote-ref-4)
4. http://users.ox.ac.uk/~atdgroup/technicalnotes/DC motor-encoder position servo controller.pdf [↑](#footnote-ref-5)
5. http://users.ox.ac.uk/~atdgroup/technicalnotes/Optical power meters for fluorescence microscopy.pdf [↑](#footnote-ref-6)
6. http://users.ox.ac.uk/~atdgroup/technicalnotes/A motorised fluorescence cube linear positioner.pdf [↑](#footnote-ref-7)
7. http://users.ox.ac.uk/~atdgroup/technicalnotes/A four position motorised optical path selector.pdf [↑](#footnote-ref-8)
8. http://freeimage.sourceforge.net/ [↑](#footnote-ref-9)
9. http://users.ox.ac.uk/~atdgroup/technicalnotes/Microscopy software architecture.pdf [↑](#footnote-ref-10)
