# Supplementary figures and images for: The Gray Institute ‘open’ high-content, fluorescence lifetime microscopes
Source: J Microsc. 2013 Jun 12;251(2):154–67. doi: 10.1111/jmi.12057 (PMC3910159; doi:10.1111/jmi.12057)

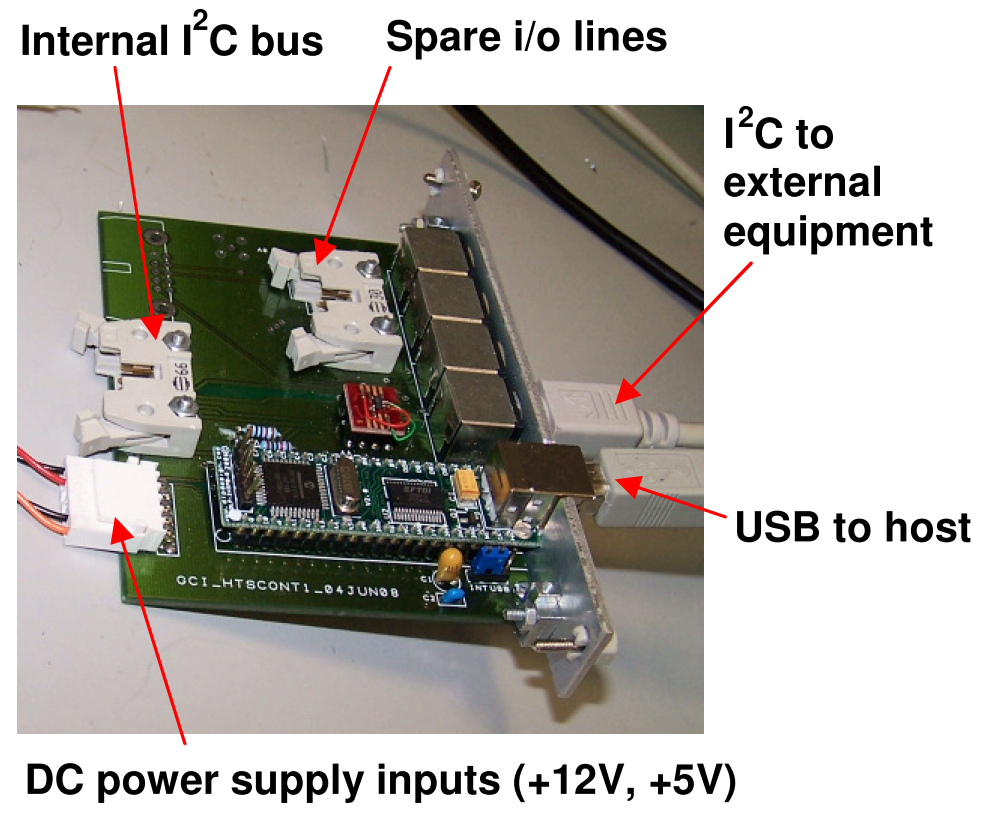

Supplement: Fig S2 — The basic assembly of the inverted microscope: two blocks attach a corner reflector mirror to the base. A fluorescence cube assembly is placed on top of this and the sample imaging objective is screwed into a combined coarse and fine adjustment focus assembly. [file jmi0251-0154-sd2.tif]

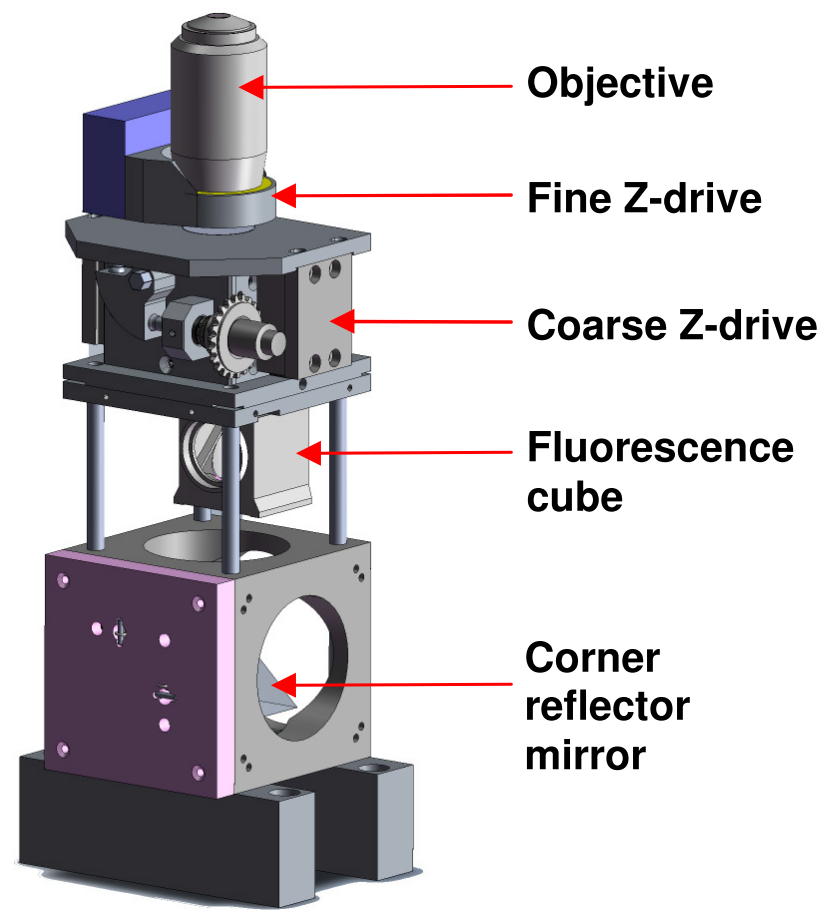

Supplement: Fig S3 — (A, C) CAD reconstruction and photograph of the dual z-drive showing an objective lens mounted onto the piezodrive. This is all atop a platform driven by a PIC controlled motor via a simple mechanical arrangement. (B, D) Software panels for controlling the coarse drive and piezo-drive. [file jmi0251-0154-sd3.tif]

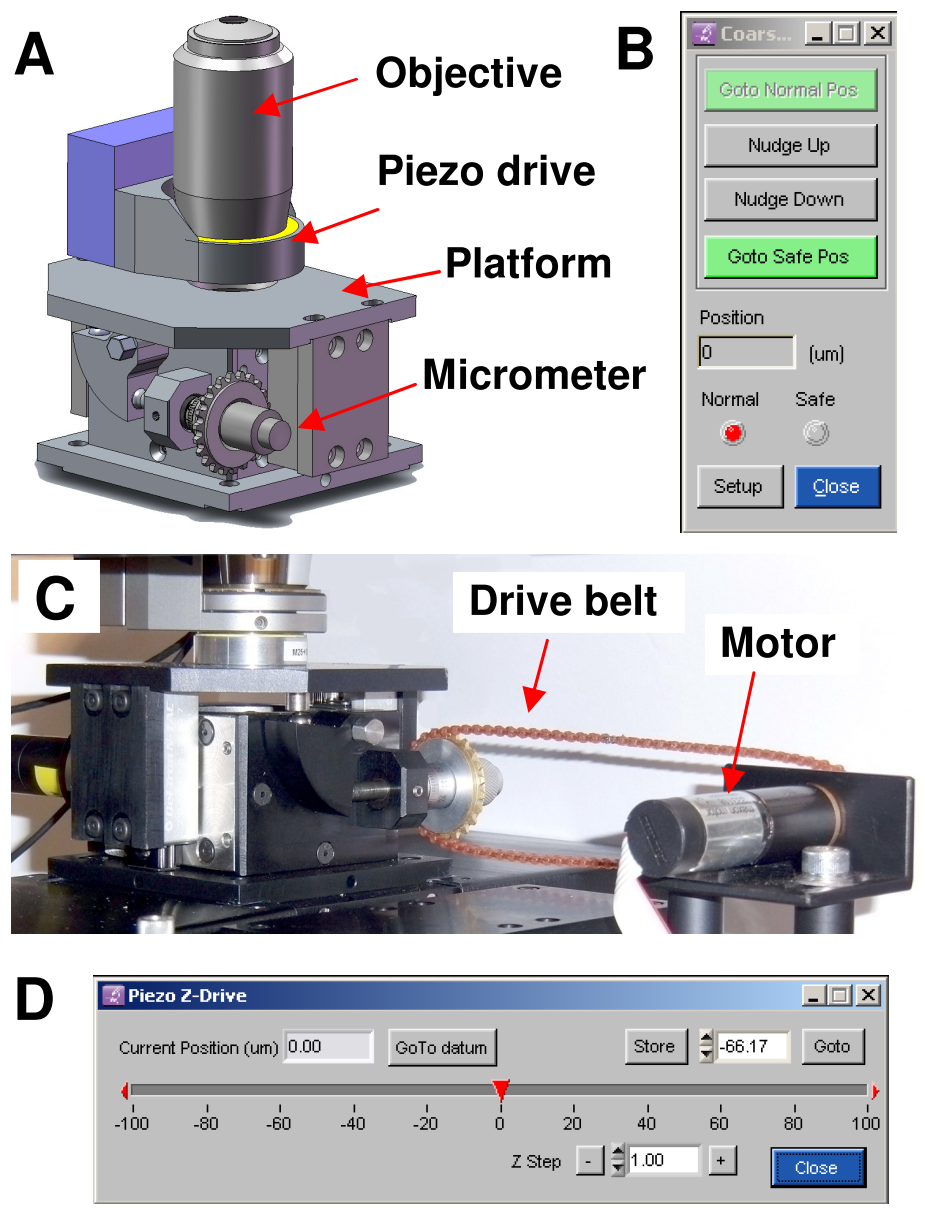

Supplement: Fig S4 — Excitation IntensityMonitor. (A) Completed viewwith cage mount ready for insertion into C4W cube. (B) Cover removed to reveal the custom compact PCB. (C) Software panel showing measured level and information about the calibration. [file jmi0251-0154-sd4.tif]

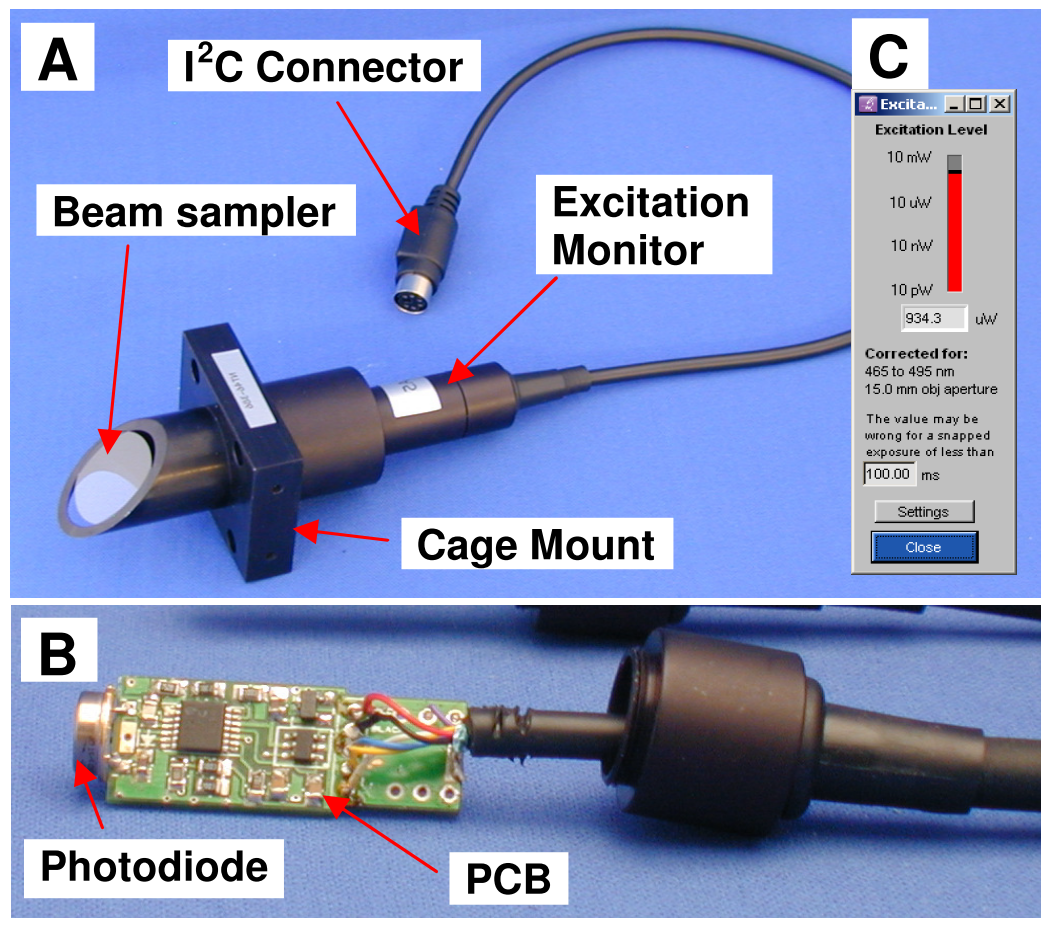

Supplement: Fig S5 — Fluorescence Filter Cube Changing Unit CAD reconstructions and photographs. (A) CAD view with transparent access covers. (B) Close-up of the cube holder (one cube of three in place). (C) Completed external view. (D) Cover removed to allow cube access. [file jmi0251-0154-sd5.tif]

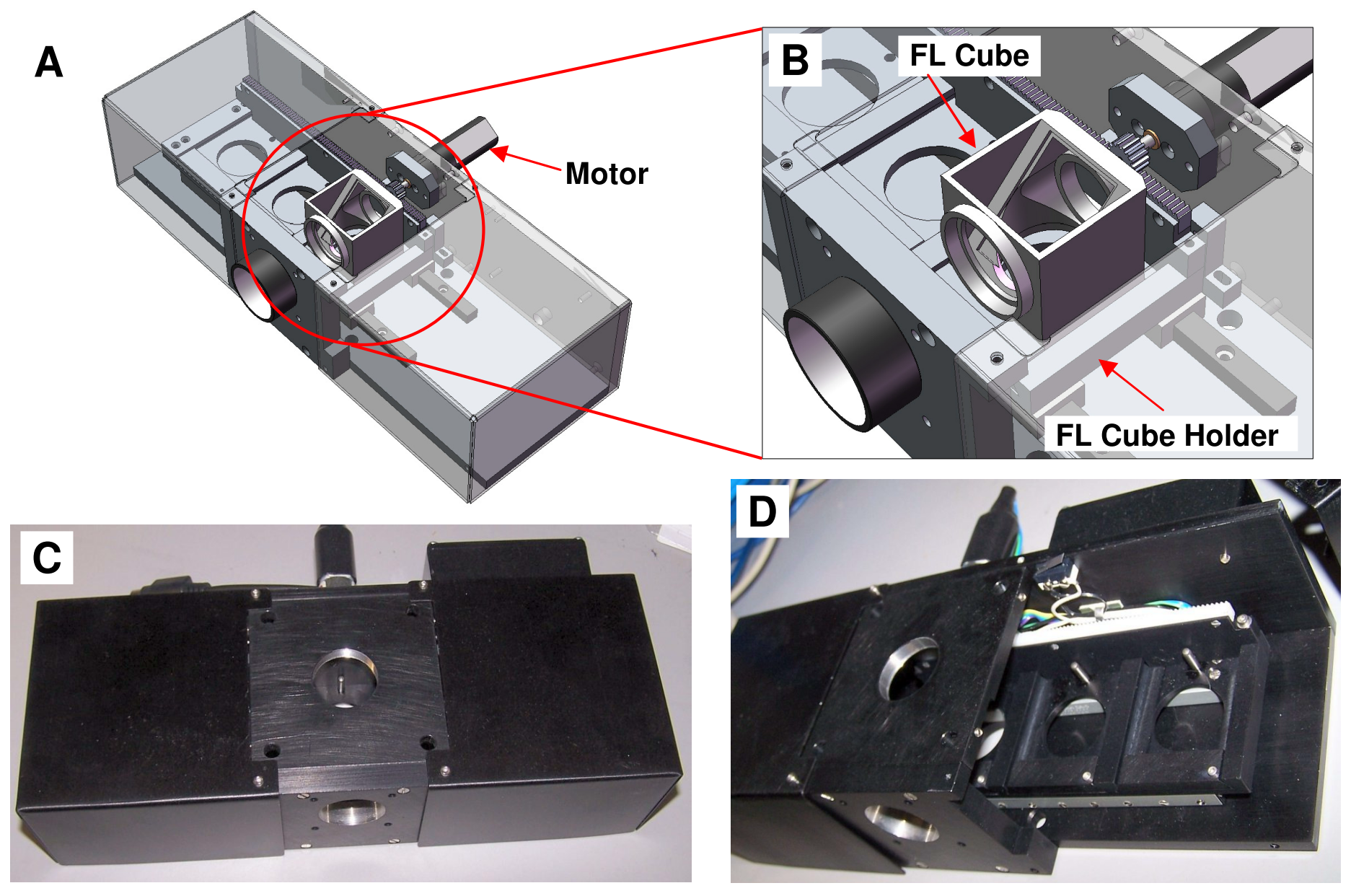

Supplement: Fig S6 — (A) A view of the four-position motorised optical path selector from the input side. (B) Exploded CAD reconstruction. [file jmi0251-0154-sd6.tif]

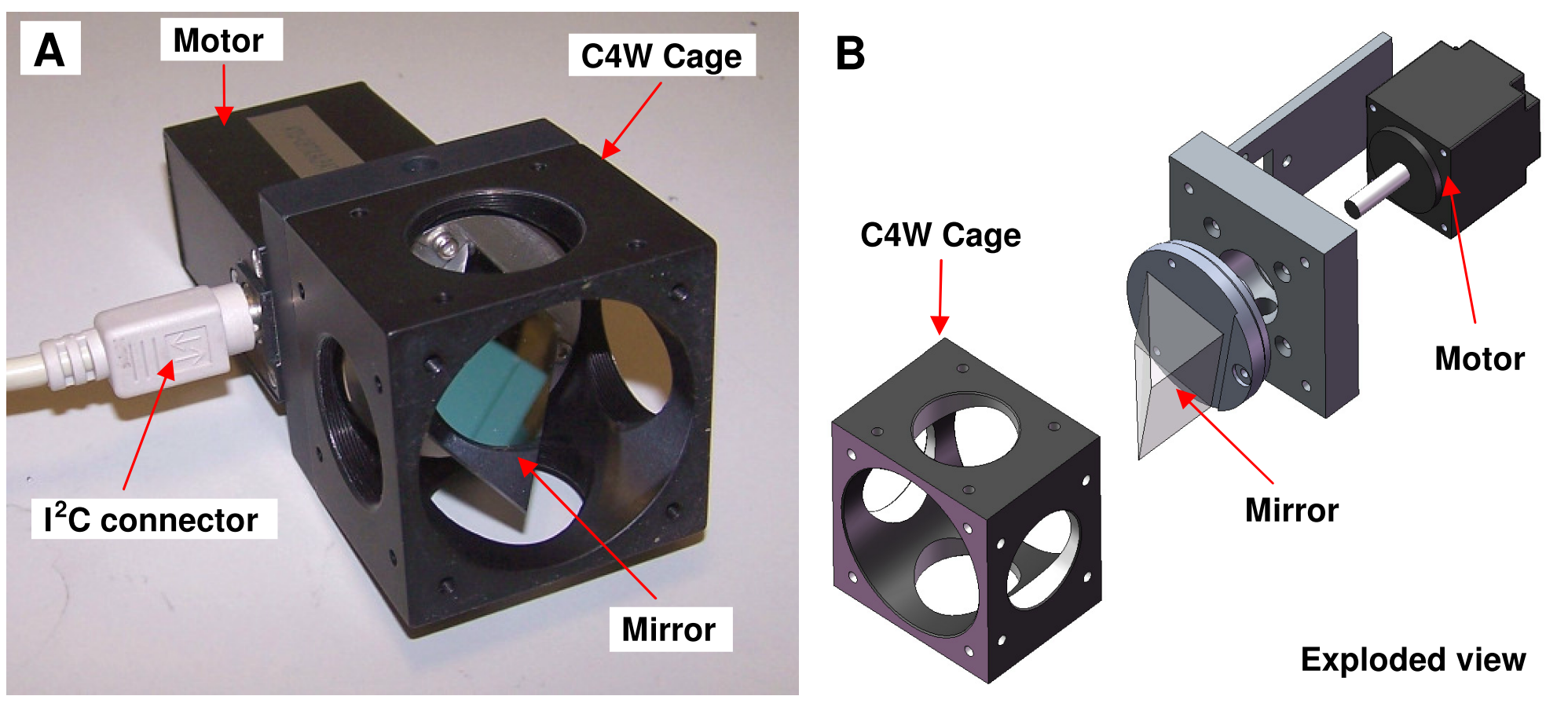

Supplement: Supplementary file 7 [file jmi0251-0154-sd7.tif]
